# Creation and study of emmer (Triticum dicoccum) × triticale hybrids

**DOI:** 10.18699/VJGB-23-39

**Published:** 2023-07

**Authors:** O.G. Silkova, Y.N. Ivanova, P.I. Stepochkin

**Affiliations:** Institute of Cytology and Genetics of the Siberian Branch of the Russian Academy of Sciences, Novosibirsk, Russia; Institute of Cytology and Genetics of the Siberian Branch of the Russian Academy of Sciences, Novosibirsk, Russia; Siberian Research Institute of Plant Production and Breeding – Branch of the Institute of Cytology and Genetics of the Siberian Branch of the Russian Academy of Sciences, Novosibirsk, Russia

**Keywords:** triticale, Triticum dicoccum, wide hybrids, genomic in situ hybridization, productivity traits, meiosis, prebreeding forms, тритикале, Triticum dicoccum, отдаленные гибриды, геномная in situ гибридизация, признаки продуктивности, мейоз, пребридинговые формы

## Abstract

Triticale (× Triticosecale Wittmack) is of great interest as an insurance crop that can ensure the stability of the gross harvest of feed and food grains at a lower cost. In Western Siberia, only winter triticale varieties are cultivated, however, spring triticales are important for cultivation in regions not suitable for winter crops. To create spring varieties with high yields and good grain quality, it is necessary to study and enrich the gene pool, identify donors of economically valuable traits. One of the possible ways to solve this problem can be through the production of secondary hexaploid triticales with the involvement of the tetraploid wild-growing species of emmer wheat Triticum dicoccum (Schrank) Schuebl. The aim of this work was to create and study hybrids of emmer T. dicoccum (Schrank) Schuebl. with hexaploid triticale using genomic in situ hybridization for staining of meiotic chromosomes and analysis of plant productivity elements in F4–F8. DT4, DT5, DT6 plants and the prebreeding F6 forms obtained from them – DT 4/168, DT 5/176 and DT 6/186 – were selected according to the characteristics of the productivity and the nature of the grain in the F4 hybrid population. The offspring of hybrids DT4 and DT5 and prebreeding forms DT 4/168 and DT 5/176 had an increased grain nature (over 750 g/l), but low productivity. The hybrid DT6 and the breeding form DT 6/186 obtained from it had high grain productivity (785 ± 41 and 822 ± 74 g/m2, respectively), but, like the paternal form of triticale UK 30/33, had a reduced nature of the grain. In F8 DT 6/186 plants, 7 homologous pairs of rye chromosomes and from 27 to 30 wheat chromosomes were found in meiosis, which indicates the presence of a complete rye genome and two wheat ААВВ genomes. Rye chromosomes showed stable formation of bivalents in contrast to wheat chromosomes, which caused the presence of aneuploids in plant populations. Thus, hexaploid forms DT 4/168 and DT 5/176 with well-made smooth grain and high grain size were obtained, which can be used as a source of this trait for selection of food-grade triticale. DT 6/186 is a promising form for further breeding in order to obtain high-yielding forms of triticale.

## Abstract (ru)

Тритикале (× Triticosecale Wittmack) представляет большой интерес как страховая культура, способная обеспечить стабильность валового сбора фуражного и продовольственного зерна с более низкими затратами. В Западной Сибири возделываются сорта только озимых тритикале, однако яровые тритикале являются значимыми для выращивания в регионах, не пригодных для озимых культур. Для создания яровых сортов с высокой урожайностью и хорошим качеством зерна необходимо изучение и обогащение генофонда, выделение доноров хозяйственно ценных признаков. Одним из возможных путей решения этой задачи может быть получение вторичных гексаплоидных тритикале с привлечением тетраплоидного дикорастущего вида пшеницы полба Triticum dicoccum (Schrank) Schuebl. Целью данной работы было создание и изучение гибридов полбы T. dicoccum (Schrank) Schuebl. с гексаплоидной тритикале с использованием геномной in situ гибридизации при окрашивании мейотических хромосом и анализ элементов продуктивности растений в F4–F8. По признакам продуктивности и натуры зерна в гибридной популяции F4 были отобраны растения ДТ4, ДТ5, ДТ6 и выявленные в их потомстве пребридинговые формы F6 – ДТ 4/168, ДТ 5/176 и ДТ 6/186. Потомства гибри- дов ДТ4 и ДТ5 и форм ДТ 4/168 и ДТ 5/176 имели повышенную натуру зерна (свыше 750 г/л), но невысокую продуктивность. Гибрид ДТ6 и полученная от него форма ДТ 6/186 отличались высокими показателями продуктивности зерна (785 ± 41 и 822 ± 74 г/м2 соответственно), но, как и отцовская форма тритикале УК 30/33, имели пониженную натуру зерна. У растений потомства F8 ДТ 6/186 в мейозе обнаружено 7 гомологичных пар хромосом ржи и от 27 до 30 хромосом пшеницы, что свидетельствует о наличии полного генома ржи и двух геномов пшеницы AABB. Хромосомы ржи демонстрировали стабильное формирование бивалентов в отличие от хромосом пшеницы, что вызвало анеуплоидию в популяциях растений. Таким образом, получены гексаплоидные формы ДТ 4/168 и ДТ 5/176 с хорошо выполненным гладким зерном и высокой натурой зерна, которые можно использовать в качестве источника этого признака для селекции тритикале пищевого направления. Форма ДТ 6/186 перспективна для дальнейшего селекционного процесса с целью получения высокоурожайных форм тритикале.

## Introduction

Triticale (×Triticosecale Wittmack) is a wheat and rye hybrid of a relatively short evolutionary history as an allopolyploid species. The first naturally created fertile wheat×rye hybrids were discovered in the late 1920s at the South-Eastern agricultural experimental station in Saratov (Meister, 1921). The plants had intermediate traits and were described as a new botanical species Triticum Secalotriticum saratoviense Meister by G.K. Meister (Levitsky, 1978). Meister immediately predicted the practical value of these intergeneric crossings. The first man-made wheat×rye hybrids (Triticosecale Wittmack) were obtained in 1888 by the German plant breeder W. Rimpau (Müntzing, 1974), who described 12 plants descending from a wheat×rye hybrid generally recognized as the first triticales (×Triticosecale Wittmack). The cytological analysis of the first triticales developed in Russia and Germany showed the somatic chromosome number of 56 (8х) (Müntzing, 1961; Levitsky, Benetzkaja, 1978), which demonstrated the combination of four genomes as follows: AABBDDRR, AABBDD, soft wheat, and RR rye.

Octoploid triticales were of great interest for plant breeders due to high seed set per spike, increased plant pathogen resistance, and resistance to environmental stresses. However, the primary triticale lines suffered from meiotic errors (Müntzing, 1961; Shkutina, Khvostova, 1971; Lukaszewski, Gustafson, 1987) and high frequency of aneuploidy in earlier generations (Krolow, 1962; Stepochkin, Vladimirov, 1978; Silkova et al., 2021), which caused reduced fertility. Grain shriveling and lateness of maturity in octoploid forms were also considered as limitations for their introduction as cultivars. As a result, there have been global efforts to develop the technology for obtaining more promising lines with various valuable breeding traits, which eventually produced hexaploid triticales (Shulyndin, 1975).

Primary hexaploid triticales of the AABBRR genome type were obtained as crossings of tetraploid wheats (T. turgidum, T. durum) and S. cereale rye (Müntzing, 1974). However, undesirable traits were not completely eliminated. Agronomically valuable traits were improved by enriching the triticale gene pool with crossing experiments involving octoploid lines and commercial soft wheat varieties, as well as hexaploid triticales with full rye genome and two wheat genomes identified in the progeny of octoploid triticales as a result of D-genome chromosome elimination (Stepochkin, 1978; Dou et al., 2006; Zhou et al., 2012; Hao et al., 2013; Li et al., 2015; Evtushenko et al., 2019). The crossing of two primary octoploid triticales produced hexaploid offspring, and breed- ing programs were focused on hybridization of these octoploids with the hexaploids identified in the progeny (Pisarev, 1964; Jenkins, 1969; Ammar et al., 2004; Oettler, 2005). Hexaploid triticales were more stable in terms of productivity (Müntzing, 1974; Oettler, 2005). As a result, various recom- binant forms of secondary triticales were derived and karyotyped (Merker, 1975; Gustafson, Bennett, 1976; Lukaszewski, Gustafson, 1983; Badaev et al., 1985; Cheng, Murata, 2002; Mergoum et al., 2009; Shishkina et al., 2009; Fu et al., 2014), and some of them having combined wheat-rye genomes showed commercial value (Merker, 1975; Oettler, 2005; Zhou et al., 2012).

Agronomic traits of triticales were further improved by intergeneric and interspecific crossings. Aegilops crassa (2n = 4x = 28; DDMcrMcr), Ae. juvenalis (2n = 6x = 42; DDCuCu MjMj), Ae. squarrosa (syn. Ae. tauschii; 2n = 2x = 14; DD) and Ae. triaristata (2n = 6x = 42; CuCuMtMtMt2Mt2) (Gruszecka et., 1996), Agropyron intermedium ssp. trichophorum (2n = 42) (Gupta, Fedak, 1986а), Hordeum parodii Covas (Gupta, Fedak, 1986b), H. vulgare L. (Balyan, Fedak, 1989) and T. monococcum L. (Neumann, Kison, 1992) plants were used in hybridization experiments with hexaploid triticales. Intergeneric polyploid triticales were also bred through hybridization with intermediate forms sharing at least one set of chromosomes (genome) with the triticale genome. These hybridization efforts produced plants with resistance genes against diseases. Lines isolated in the progeny of triticale hybrids (AABBRR) and amphidiploids (wheat × Psathyrostachys huashanica, 2n = 8x = 56, AABBDDNsNs) were resistant to yellow rust (Kang et al., 2016). Hybridization of a hexaploid triticale and an amphiploid intermediate form (Ae. variabilis × S. cereale, 2n = 6x = 42; UUSvSvRR) produced addition and substitution lines with the 3Sv Ae. variabilis chromosome carrying the powdery mildew resistance gene Pm13 (Kwiatek et al., 2016). Addition and substitution lines for chromosome 2D with the leaf-rust resistance gene Lr39 and semidwarf gene Rht8, as well as chromosome 3D (or 3D/3B) with the Lr32 gene were isolated in the progeny of triticale × amphiploid hybrids (Ae. tauschii × S. cereale, 2n = 4x = 28; DDRR) (Kwiatek et al., 2015).

Thanks to breeding achievements, triticale has become a new economically significant cereal species characterized by high grain and vegetative mass productivity, that could be used as forage and green feed (McGoverin et al., 2011; Ayalew et al., 2018). In the last three decades, its products have become increasingly significant, which is demonstrated by increasing crop acreage across the world, from 1,453,269 ha in 1994 to 4,157,018 ha in 2016. Triticale grains are used to produce bioethanol and food wrap, as well as various food products (bread, biscuits, pastas, flatbreads, and malt) (Zhu, 2018), and bran is used as the source of prebiotics and antioxidants for yoghurts. Food-grade triticale grains are comparable to wheat in macro- and micronutrient contents (Zhu, 2018). Protein content in triticale grains is 1–1.5 % higher than in wheat and 3–4 % higher than in rye, gluten content matches that in wheat or is 2–4 % higher (Meleshkina et al., 2015). However, triticale underperforms in test weight. This parameter is closely related to the plumpness and hardness of grains, as well as to their size and shape. Average test weight for wheat is 700–810 g/l. At test weights below 740 g/l, flour yield tends to decrease rapidly as test weight goes down. Most spring triticales have shriveling grains and low flour yields, which limits their use in bread making (Rakha et al., 2011).

Development of domestic high-productivity triticale varieties with high grain quality requires further study and enrichment of the gene pool, as well as identification of donors for economically valuable traits. One way to solve this problem is to obtain secondary hexaploid triticales using emmer wheat T. dicoccum (Schrank) Schuebl., a tetraploid wild-growing wheat with long, large, and plump grains.

The goal of the present study was to breed new forms of hexaploid triticales (ААВВRR genome type) with improved test weights by crossing emmer (T. dicoccum Schrank, ААВВ genome) with triticale and study their productivity and meiotic stability with chromosome staining using genomic in situ hybridization.

## Materials and methods

Plant material. New forms of hexaploid triticale were obtained by hybridization of emmer (T. dicoccum (Schrank) Schuebl.) and triticale (×Triticosecale Wittmack). Intergeneric F1 hybrid of emmer lines (L133 × PKK) × k-25516 (AABB genome) was used as maternal plants. Awned semi-hulless emmer (L133 × PKK) created by VIR researchers is characterized by brittle spikes and low productivity, and awnless emmer k-25516 was obtained at Siberian Research Institute of Plant Production and Breeding (SRI PPB), ICG SB RAS from the population of awned emmer wheat from the VIR collection. The paternal plants were represented by a selection form of hexaploid triticale UK 30/33 selected from the population of cytogenetically unstable octoploid triticale UK30 (AABBDDRR genome) developed at the SRI PPB by crossing the Ulyanovka soft wheat variety with the Korotkostebelnaya 69 short stem rye with subsequent doubling of chromosome number induced by colchicine water solution.

The progeny of three F4 hybrids DT4, DT5, and DT6, and three selection forms DT 4/168, DT 5/176, and DT 6/186 isolated in F5 from hybrid populations DT4, DT5, and DT6 respectively was selected for the study. In 2020, their F6 progeny was seeded for research purposes along with F4 hybrids at the nursery for distant wheat hybrids in the field of SRI PPB.

Fluorescence in situ hybridization of meiotic chromosomes. To evaluate the meiotic stability in the selection forms, two most productive plants DT 6/186/156 and DT 6/186/165 were selected in the F7 progeny of plant DT 6/186, and their F8 seeds were seeded in the hydroponic greenhouse of the ICG SB RAS in spring 2021. The chromosomal behavior of the progeny was studied using routine acetocarmine staining protocol and FISH staining (fluorescence in situ hybridization) following the technique described earlier (Ivanova et al., 2019).

The meiocytes were analyzed at the diakinesis stage, metaphase I (МI), anaphase I (AI), and telophase II (TII). The probes used in the analysis were as follows: Aegilops tauschii pAet6-09 specific for centromere repeats in rice, wheat, rye, and barley chromosomes (Zhang et al., 2004); pAWRc specific for centromere repeats in rye chromosomes (Francki, 2001), and rye genomic DNA. DNA repeat samples of pAet6- 09 and pAWRc were the courtesy of Dr. A. Lukaszewcki (University of California, Riverside, the United States). Centromere-specific probes were labeled with biotin 16-dUTP or digoxigenin 11-dUTP by means of polymerase chain reaction (PCR). The total rye DNA was labeled by Nick translation with digoxigenin 11-dUTP. The probes were combined in different proportions and mixed with blocking wheat DNA. The preparations were mounted in Vectashield antifade solution (Vector Laboratories) slowing down fluorescence fading and including 1 μg/ml DAPI (4′,6-diamidino-2-phenylindol, Sigma-Aldrich, the United States) for chromatin staining. All preparations were analyzed using an Axio Imager M1 microscope (Karl Zeiss, Germany), the images were recorded using a ProgRes MF camera (Meta Systems, Jenoptic) at the Center of Microscopic Analysis of Biological Objects, SB RAS and processed using Adobe Photoshop CS2 software.

Analysis of economically valuable traits. Structural analysis of the plants was performed at the facility equipped for metric measurements, threshing, and seed weighting. As a result, the following data on productivity elements were obtained: spike length; spike density; grain weight per spike; thousand kernel weight; grain number per spikelet; test weight measured using a microchondrometer (Stepochkina, Stepochkin, 2015); and grain productivity per 1 m2. Statistical processing of the results was carried out following the standard technique (Dospekhov, 1985). Significance of differences between mean values of two samples was estimated using Student’s t-test.

## Results

**Fig. 1.**
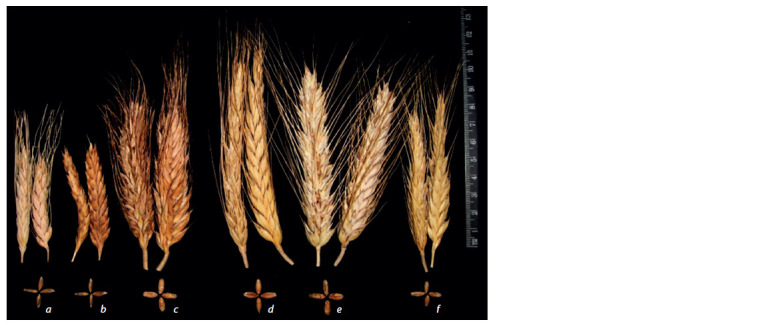
Spikes and seeds: a, emmer wheat (L133 × PKK); b, emmer wheat k-25516; c, triticale UK 30/33; F4 hybrids: d, DT4; e, DT6; f, DT5.

The parental-line plants had different spike and grain morphology. The emmer plants (L133 × PKK) had short, awned, brittle spikes and smooth, long grains (Fig. 1, a). The k-25516 emmer sample was an awnless plant with thin, long grains (see Fig. 1, b). Triticale UK 30/33 had a dense, awned spike and grains similar to those of soft wheat in shape but shriveling (see Fig. 1, c).

Plants of three emmer×triticale F4 hybrids had different spike morphology (Table 1, see Fig. 1, d–f ). The DT6 hybrid had a dense, awned spike. The plants of the remaining two hybrids had looser spikes. The spikes were sterile at the ends and therefore often susceptible to ergot.

**Table 1.**
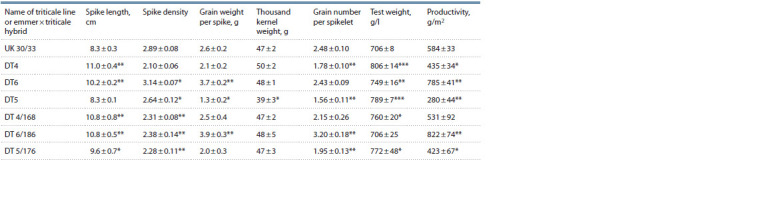
Results of structural analysis of triticale plants and emmer × triticale hybrids p 0.05; ** p < 0.01; *** p < 0.001 – significant differences between the hybrid and 6х triticale UK 30/33.

All hybrids had a hair neck similar to the paternal triticale UK 30/33. This means that these genotypes have a gene responsible for manifestation of this trait localized in the long arm of chromosome 5R.

Triticale UK 30/33 had a short spike, low test weight, and medium grain productivity (see Table 1). Selection forms and hybrids, except for DT5, had a longer spike than the paternal form UK 30/33. Hybrid DT5 had low productivity due to flower sterility in the upper part of the spike and low thousand kernel weight. Hybrid DT4 had a high thousand kernel weight of 50 ± 2 g, and the highest test weight of 806 ± 14 g/l. However, its grain productivity, although higher than that of DT5, was still not too high due to low seed set of spikes and spikelets. Flowers at the top of the spike were often sterile as well. Hybrid DT6 was characterized by dense spikes and high grain productivity (785 ± 41 g/m2), as well as seed set of spikes and spikelets. The spikes were fertile along their full length.

Selection forms DT 4/168, DT 5/176, and DT 6/186 had different grain weights per spike, grain numbers per spike, test weights, and productivities. DT 4/168 had denser spikes and slightly higher seed sets of spikes and spikelets compared to the DT4 hybrid it was obtained from. Breeding sample DT 5/176 was more productive than the initial DT5 hybrid, but it had the lowest productivity among the three breeding samples studied. Small spike size and sterility of 3–7 spikelets in the upper part of the spike resulted in low productivity of hybrids DT5 and DT 5/176. The DT 6/186 line had the highest seed set and productivity among the studied lines. Despite the lowest test weight values similar to triticale UK 30/33 (706 ± 25 g/l), grain productivity per plot reached 822 ± 74 g/ m2. DT 6/186 had a smoother overall morphology than the initial hybrid DT6, but higher breeding value due to its higher grain productivity.

**Fig. 2.**
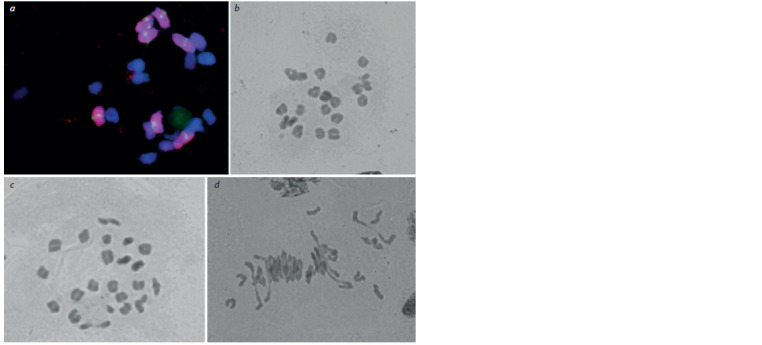
Diakinesis (a–c) and metaphase I (d ) meiotic stages in the progeny of DT 6/186/156 plant (a, b) and DT 6/186/165 plant (c, d ). a, b, 21 bivalents, a, 7 rye bivalents; c, 21 bivalents and one univalent; d, univalents in metaphase I. a, genomic in situ hybridization, rye chromosomes stained red; b–d, acetocarmine staining.

**Fig. 3.**
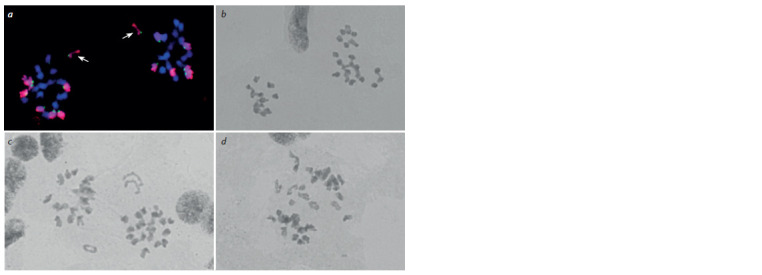
Chromosome separation anomalies in meiotic anaphase I in the progeny of DT 6/186/165: a, unequal separation of wheat and rye chromosomes, the rye chromosome is divided into sister chromatids (arrows); b, unequal chromosome separation; c, d, lagging chromosomes at the equator and univalent division into sister chromatids. а, genomic in situ hybridization, rye chromosomes stained red; b–d, acetocarmine staining.

Cytological analysis of the progeny of two plants (DT 6/186/ 156 and DT 6/186/165) from the highly productive DT 6/186 line revealed instability in chromosome number and errors in chromosomal behavior in the first and second meiotic divisions. Chromosome staining using genomic in situ hybridization in these plants showed 14 rye chromosomes forming bivalents, which implied the presence of seven homologous pairs (Fig. 2, a). However, the bivalent chromosomes demonstrated premature separation in some meiocytes (desynapsis), with rye chromosomes becoming univalent and distributing anomalously between the poles (Fig. 3, a).

Chromosome number in the discovered aneuploid plants varied from 2n = 41 to 2n = 44. Among the ten plants from the DT 6/186/156 progeny, only one plant had chromosome number 2n = 42 (see Fig. 2, b), while there were no plants with euploid chromosome numbers in the progeny of DT 6/186/165.

**Fig. 4.**
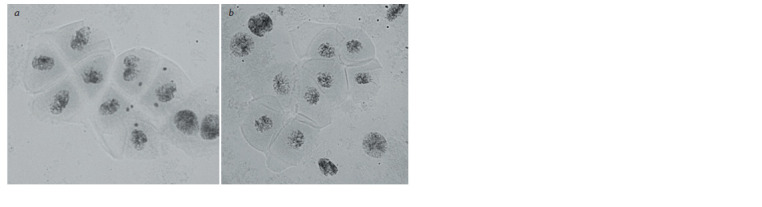
Tetrads with (a) and without (b) micronuclei

Univalents were discovered in the metaphase I (see Fig. 2, d ), which were lagging at the equator during chromosome separation in anaphase I in 86.75 ± 4.56 and 61.32 ± 2.81 % of cells in DT 6/186/156 and DT 6/186/165 respectively (Table 2, see Fig. 3, c, d ). The lagging chromosomes were divided into sister chromatids (see Fig. 3, a, c, d )or broken at the centromere. Instability of chromosome separation during the division resulted in micronuclei formation at the tetrad stage (Fig. 4, a). 

**Table 2.**
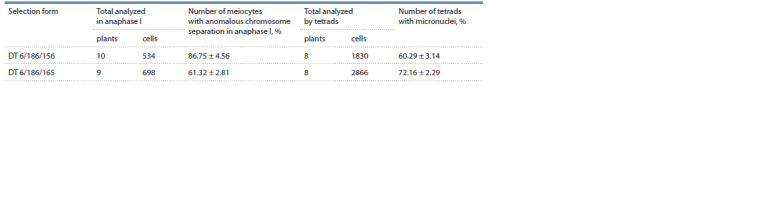
Analysis of meiotic chromosomal behavior in F8 plants

Micronuclei were observed in 60.29 ± 3.14 and 72.16 ± ± 2.29 % tetrads in DT 6/186/156 and DT 6/186/165, respectively (see Table 2). Even the euploid plant with 2n = 42 (DT 6/186/156) had micronuclei in 51.48 % tetrads, which prevented us from considering this plant fully cytogenetically stable.

Plants with the minimum number of anomalous meiocytes in anaphase I and telophase II were selected from populations DT 6/186/156 and DT 6/186/165 based on the results of the analysis.

## Discussion

Triticale (× Triticosecale Wittmack) as an agricultural crop combines wheat’s high yield potential with rye’s resistance to biotic and abiotic stresses, which increases its adaptability to cultivation conditions in salty or highly acidic soils and in presence of toxic heavy metals. Thanks to these traits, triticale is of great interest as an emergency crop ensuring stable gross harvest of forage and food grains at lower costs (McGoverin et al., 2011). Despite today’s applications of triticale grains being mostly restricted to forage in animal husbandry and production of feed and bioethanol, there is a rising interest in the use of triticale grains in human food products. Triticale grains have proven nutritional and dietary value (Meleshkina et al., 2015; Zhu, 2018), since they include not only proteins, carbohydrates, and fats, but also vitamins, minerals, and dietary fibres (14–18 %) (Rakha et al., 2011; Zhu, 2018). Compared to that of wheat, the protein from its grains has a richer amino acid profile, particularly in indispensable amino acids, such as lysine, threonine, and leucine (Meleshkina et al., 2015; Torikov et al., 2019). Triticale starch amounting to 3/4 of the kernel weight has a significantly lower amylose content compared to rye and wheat (Zhu, 2018), which ensures its better digestion by humans (Meleshkina et al., 2015).

To increase the share of triticales in production of breads and pastries, in recent decades breeding efforts have been aimed at increasing the quality of grains and finished products, which has resulted in development of triticale bread-making varieties (Grabovets et al., 2013), so State standards for triticale flour have been developed (State Standard 34142-2017). The characteristics of winter triticale varieties also include applicability for bread making. Triticale breeding efforts in Russia and other countries are primarily aimed at developing winter varieties (State Register…, 2022). However, grain quality assessment of the samples from the spring triticale collection showed that spring triticales have good potential for creating bread-making varieties (Krokhmal, Grabovets, 2015; Bocharnikova et al., 2017; Abdelkawy et al., 2020; Yerzhebayeva et al., 2020). The samples with such improved parameters as protein content, test weight, falling number, vitreousness, gluten quantity and quality, etc., are used in breeding for improved grain quality (Krokhmal, Grabovets, 2015; Bocharnikova et al., 2017; Turbayev et al., 2019; Abdelkawy et al., 2020; Yerzhebayeva et al., 2020).

A trait such as high test weight can be transferred by distant hybridization. This parameter is closely related to genetically determined parameters such as grain plumpness, hardness, and shape. In the present paper, tetraploid emmer wheat T. dicoccum (Schrank) Schuebl. with long, large, and plump grains was used as the maternal line for hybridization with hexaploid triticale (× Triticosecale Wittmack). As a result, new hexaploid forms of triticale with AABBRR genome types were created, which is confirmed by the analysis of meiotic chromosomal behavior using genomic in situ hybridization. Seven pairs of rye chromosomes and 27 to 30 wheat chromosomes were observed in the plants, which implies the presence of a complete rye genome and two wheat genomes. Three plants, DT4, DT5, and DT6, were isolated in the F4 population, the progeny of which demonstrated test weight values significantly exceeding those of the initial triticale line, and test weights in the progeny of DT 4/168, DT 5/176, and DT 6/186 forms from F6 were higher or on par with those of the initial triticale line. These plants had different productivity values, with the highest ones in F4 recorded in the DT6 line (785 ± 41 g/m2). The respective value for DT 6/186, i. e., the progeny of the latter in F6, reached 822 ± 74 g/m2. The DT 4/168 and DT 5/176 lines are of moderate interest for further research into improvement of bread-making properties of triticale grains.

The study of the meiotic behavior of rye and wheat chromosomes in the progeny of F8 plants DT 6/186/156 and DT 6/186/165 showed that they had not yet achieved cytogenetic stability, which was indicated by the discovered chromosome separation errors and the presence of aneuploids in the populations. Chromosome separation errors mostly occurred in wheat chromosomes due to their monosomy. Cytological instability and aneuploidy in octoploid and hexaploid wheatrye allopolyploids have been an issue from the start (Shkutina, Khvostova, 1971; Kaltsikes, 1974; Weimarck, 1974; Lukaszewski, Gustafson, 1987), but secondary triticales turned out to be more cytogenetically stable than primary ones (Kaltsikes, 1974). Cytological study of triticales showed that the interaction between wheat and rye genomes in the cells of the same plant resulted in physiological defects in cells persisting for decades at least. Meiotic and mitotic errors were found both in the triticale obtained by Rimpau in 1888 (Levitsky, 1978; Müntzing, 1974) and in the triticales obtained later. Despite the complete set of chromosomes, meiotic univalents were found in triticales with different ploidy levels (Shkutina, Khvostova, 1971; Kaltsikes, 1974; Lukaszewski, Gustafson, 1987; Olesczuk, Lukaszewski, 2014; Orlovskaya et al., 2015). The study into meiotic chromosomal behavior in triticales in the present and earlier papers demonstrated that while only bivalents were present at the diakinesis stage, univalents appeared in metaphase I as a result of to produce lagging chromosomes at the equator (Shkutina, Khvostova, 1971). Presumably, aneu- ploid cells in triticales may emerge as a result of asynchronous separation of rye and wheat chromosomes and their lagging in anaphase and telophase (Shkutina, Khvostova, 1971). The presumed cause of meiotic instability in the obtained amphidiploids is an imbalance in the genetic system of meiotic pairing and the differences in cell cycles between wheat and rye (Müntzing, 1974).

It is known for a fact that chromosome separation depends on kinetochore function (Sanei et al., 2011). It was found that kinetochore protein CENH3 produced by one of the parents maintained the function of other parental kinetochores in stable hybrids, despite the differences between DNA sequences of centromere regions in parental lines (Ishii et al., 2016). It was also shown that cytogenetic stability in triticales could also be linked to increased expression of rye-specific CENH3 forms in a hybrid genome (Evtushenko et al., 2019).

Another possible cause of cytogenetic instability of the obtained triticales could be a nuclear-cytoplasmic incompatibility, because the hybrids are bred using an intergeneric F1 hybrid of emmer lines (T. dicoccum (Schrank) Schuebl.) (L133 × PKK) × k-25516 (genome AABB) as the maternal form. The AABB genomes in paternal triticale forms originate from soft wheat, while the rye genome is used as the base. Selection of genotypes of wheat varieties during triticale backcrossing may lead to recovery of fertility and cytogenetic stability in new forms, as exemplified by alloplasmic soft wheat lines (H. vulgare)-T. aestivum (Pershina et al., 1999, 2018; Trubacheeva et al., 2021).

## Conclusion

The result of this work was to create and study hybrids of emmer T. dicoccum (Schrank) Schuebl. with hexaploid triticale. According to the characteristics of productivity and test weight values of the grain, hexaploid prebreeding forms F6 were isolated – DT 4/168, DT 5/176 and DT 6/186. Thus, hexaploid forms DT 4/168 and DT 5/176 with well-filled, smooth grains and high test weights that can be used as the source of said traits in food-grade triticale breeding have been obtained. The DT 6/186 line shows promise in terms of breeding for high yields. As a result of the analysis of meiotic division in DT 6/186 plants, this sample is unstable for wheat chromosomes, therefore, aneuploids will occur in the offspring. To restore fertility and cytogenetic stability of new forms of triticale with emmer cytoplasm and chromosomes, it is necessary to select the genotypes of wheat varieties during backcrossing

## Conflict of interest

The authors declare no conflict of interest.
